# Urapidil as a neuroprotective agent: targeting hypoxia, inflammation, and oxidative stress in traumatic brain injury

**DOI:** 10.1007/s00068-025-02873-z

**Published:** 2025-05-06

**Authors:** Ahmet Bindal, Pınar Karabacak, Halil Asci, Ilter Ilhan, Muhammet Yusuf Tepebasi, Mehmet Abdulkadir Sevuk, Orhan Imeci, Ozlem Ozmen, Burak Yildirim

**Affiliations:** 1 Department of Anesthesiology and Reanimation, Faculty of Medicine, Suleyman Demirel University, Isparta, Türkiye; 2 Department of Pharmacology, Faculty of Medicine, Suleyman Demirel University, Isparta, Türkiye; 3 Department of Biochemistry, Faculty of Medicine, Suleyman Demirel University, Isparta, Türkiye; 4 Department of Genetic, Faculty of Medicine, Suleyman Demirel University, Isparta, Türkiye; 5 Department of Pathology, Faculty of Veterinary Medicine, Burdur Mehmet Akif Ersoy University, Burdur, Türkiye

**Keywords:** Apoptosis, Inflammation, Neuroprotection, Rat, Trauma

## Abstract

**Purpose:**

One of the important causes of morbidity and mortality in the world is traumatic brain injury (TBI), which is a process that triggers damaging mechanisms such as inflammation, oxidative stress, and apoptosis. The results of current pharmaceutical methods are not enough, and researches into new therapy modalities are needed. This study aimed to evaluate the neuroprotective effects of Urapidil (Ura), which is an alpha-1 adrenergic receptor antagonist with serotonergic activity, in a TBI model and investigating signaling pathways like high mobility group box 1 (HMGB1), BCL2-interacting protein 3-like (BNIP3L), and hypoxia-inducible factor-1 alpha (HIF1α).

**Methods:**

Thirty-two rats were divided into four groups: control, TBI, TBI + Ura_0.5_ (0.5 mg/kg), TBI + Ura_5_ (5 mg/kg) groups. Tissue integrity and expressions of tumor necrosis factor-alpha (TNF-α), caspase-3 (Cas-3), tyrosine hydroxylase (TH), HIF1α, BNIP3L, and HMGB1 were assessed. Ura’s biochemical oxidative stress indicators were also assessed.

**Results:**

Ura treatment at both doses, significantly decreased histopathological findings, BNIP3L, HMGB1, and HIF1α expressions, TNF-α, Cas-3, TH immunexpressions, and TOS and OSI levels, and elevated TAS levels compared to TBI group. These results show that Ura regulates molecular pathways related to TBI, including neuroinflammation, mitochondrial dysfunction, and hypoxia.

**Conclusion:**

Ura shows promising tissue-protective effects in TBI by targeting inflammation, oxidative stress, and apoptosis. This study provides a new perspective on the need for further development of Ura for therapeutic use.

## Graphical Abstract

**Figure:**
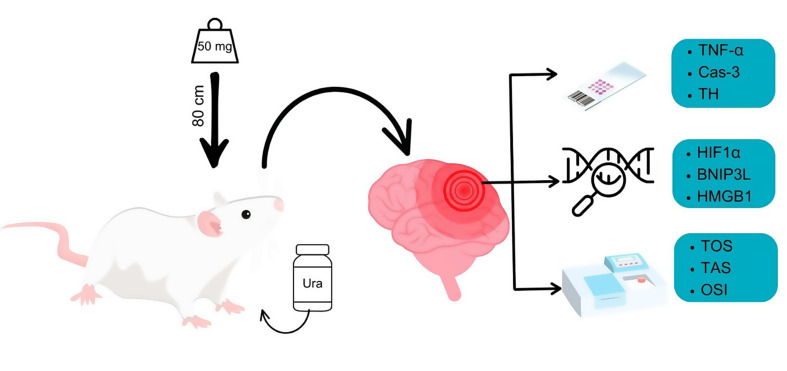

## Introduction

Traumatic brain injury (TBI) is a worldwide health issue that develops after original mechanical damage and advances through intricate secondary damage mechanisms such as oxidative stress, inflammation, and apoptosis [1]. TBI is one of the main causes of morbidity and mortality globally due to these degenerative processes, which produce extensive brain damage and functional impairments [2]. Although the pathophysiology of TBI has advanced significantly, there is still no proven pharmacological treatment for the condition, and there are few effective treatment approaches that target the subsequent injury cascade. This unmet clinical need emphasizes how crucial it is to investigate new treatment agents that could lessen the damage caused by TBI and enhance neurological results.

The importance of molecular regulators such as high mobility group box 1 (HMGB1), BCL2-interacting protein 3-like (BNIP3L), and hypoxia-induced factor-1 alpha (HIF1α) in the development of TBI has been highlighted in recent years [3, 4]. A crucial modulator of the physiological response to hypoxia, HIF1α is markedly elevated following TBI and plays a role in neuroinflammation and apoptotic cell death [5]. One element that worsens mitochondrial dysfunction and initiates programmed cell death is BNIP3L. However, HMGB1 is a potent pro-inflammatory regulator that exacerbates tissue damage and neuroinflammation [6]. Developing effective therapeutic strategies for TBI can be facilitated by focusing on these biological markers.

In several experimental models, Urapidil (Ura), an alpha-1 adrenergic receptor antagonist that can also affect serotonergic receptors, has shown anti-inflammatory, antioxidant, and anti-apoptotic properties [7]. Ura may be a neuroprotective drug that alters important molecular pathways linked to TBI because of its adaptable pharmacological profile. This study uses histological, immunohistochemical, and genetic investigations to examine the therapeutic effectiveness of Ura in the rat TBI model. Its effects on biochemical indicators of oxidative stress, specifically HIF1α, BNIP3L, and HMGB1 expressions, are assessed. The goal of this research is to better understand the pathophysiology of TBI and aid in the development of targeted therapeutics by clarifying the processes underlying Ura’s protective effects.

## Materials and methods

### Ethical approval

The procedures conducted on rats were subjected to review and approval by the Animal Experiments Local Ethics Committee of Suleyman Demirel University (Ethic No: 11.05.2023-05/167). The experiment adhered to the Animal Research: Reporting of In Vivo Experiments (ARRIVE) 2.0 guidelines. Furthermore, support for the study was provided by the Suleyman Demirel University Scientific Research Project Unit under project number TSG-2023-9092.

### Animals

Thirty-two adult male Wistar albino rats (weighing 300–350 g) were housed in standard Euro-type 4 cages, with groups kept separate. The animals were maintained under controlled conditions at 23 °C and 55% humidity, following a 12-hour light/dark cycle. They were provided with standard commercial feed and water ad libitum.

Head trauma was induced by using the Marmarou impact-acceleration model. A 50 mg ball was dropped from a height of 80 cm, generating a trauma force of approximately 0.2 N as per Newton’s law [8]. The experimental groups were designed as follows:

1. Control group: Rats received an intraperitoneal (i.p.) injection of 0.5–1 mL saline (SF) without undergoing trauma. After 24 h, the animals were sacrificed under anesthesia, and brain tissues were collected.
2. TBI group: Under anesthesia, trauma was induced, followed by an i.p. injection of 0.5–1 mL SF 30 min later. After 24 h, the animals were sacrificed under anesthesia, and brain tissues were collected.
3. TBI + Ura_0.5_ group: Following trauma induction under anesthesia, rats received 0.5 mg/kg Ura i.p. 30 min later [9]. After 24 h, the animals were sacrificed under anesthesia, and brain tissues were collected.
4. TBI + Ura_5_ group: Rats were subjected to trauma under anesthesia and administered 5 mg/kg Ura i.p. 30 min post-trauma. After 24 h, the animals were sacrificed under anesthesia, and brain tissues were collected.

For both trauma induction and sacrifice, rats were anesthetized using 10 mg/kg xylazine (Xylazin Bio 2%, Bioveta, Czech Republic) and 90 mg/kg ketamine (Keta-Control, Doğa İlaç, Turkey). Sacrifice was performed via surgical exsanguination by drawing blood from the inferior vena cava. Blood samples and brain tissues were collected after sacrification. Half of the brain tissues were fixed in formaldehyde for histopathological and immunohistochemical evaluation, while the remaining tissues were stored in tubes at -80 °C for biochemical and genetic analyses.

### Histopathological analyses

After being gently removed, the brains were fixed in a 10% buffered formalin solution during the necropsy. After a 2-day fixation, the brain samples were trimmed and placed into tissue processing cassettes. The tissues underwent routine tissue processing and were then embedded in paraffin. Hematoxylin-eosin (HE) staining was applied to the brain samples after the paraffin blocks were cut into 5 μm thick sections. The tissues were examined histopathologically using the Olympus CX41 light microscope. Morphometric analyses and microphotography were carried out using the Database Manual Cell Sens Life Science Imaging Software System (Olympus, Tokyo, Japan).

The histopathological data were evaluated using the criteria listed in Table 1, similar to the approach used in the study by Mielke et al. [10]. Possible scores ranged from 0 to 4.

**Table 1:** Histopathological scores of subarachnoid hemorrhages

0	Normal meningeal and parenchymal structure
1	No blood in the subarachnoid space, ventricles, or brain parenchyma.
2	No localized or diffuse thin subarachnoid hemorrhage, intraventricular, or intraparenchymal hemorrhage.
3	No diffuse or localized thick subarachnoid blood layers, intraventricular, or intraparenchymal hemorrhage.
4	Intraventricular or intraparenchymal hemorrhage in association with subarachnoid hemorrhage, regardless of thickness or location.

### Immunohistochemical method

Sections mounted on poly-L-lysine-coated slides underwent immunohistochemical staining using the streptavidin-biotin peroxidase method. Primary antibodies targeting tyrosine hydroxylase (Recombinant Anti-Tyrosine Hydroxylase antibody [EP1532Y] - Neuronal Marker (ab137869)), Caspase-3 (Cas-3) (Anti-Cas-3 antibody [EPR18297] (ab184787)), and tumor necrosis factor-alpha (TNF-α) (Recombinant Anti-TNF-α antibody [EPR21753-109] (ab205587)) (Abcam, Cambridge, UK) were employed for the immunohistochemical analysis of brain sections. All primary antibodies were diluted at a ratio of 1:100 using antibody dilution solutions. The immunohistochemistry process was conducted according to the manufacturer’s instructions. The Mouse and Rabbit Specific HRP/DAB Detection Kit - Micropolymer (ab236466) (Abcam, Cambridge, UK) was utilized as the secondary kit in this investigation. For negative controls, antibody dilution solutions were applied to the sections at the primary antibody stage instead of primary antibodies.

During the immunohistochemical analysis, the percentage of positive cells was quantified and compared across the groups. Special attention was given to examining cells from both control groups and brain regions affected by trauma. For this purpose, 20 randomly selected cells from each of five areas within the same brain region were counted for each rat using a 40X objective, resulting in a total of 100 cells per area. The number of cells exhibiting a positive immunohistochemical reaction was determined using Image J 1.48 software (National Institutes of Health, Bethesda, MD). The results were captured using an Olympus CX41 microscope, and microphotography was performed using the Database Manual Cell Sens Life Science Imaging Software System (Olympus Corporation, Tokyo, Japan).

### Biochemical analysis

Rat brain tissues (approximately 150 mg each) were homogenized in a 1:9 (w/v) phosphate-buffered saline solution (pH 7.4) using the Ultra Turrax homogenizer (IKA^®^ Werke, Germany). After homogenization, the samples were centrifuged at 10,000 rpm for 10 min to assess oxidative stress parameters. Total oxidant status (TOS), total antioxidant status (TAS), and oxidative stress index (OSI) levels were analyzed in the homogenized tissue samples using an automated analyzer and Erel’s colorimetric method (Beckman Coulter, USA). The OSI value was calculated using the formula OSI = [(TOS, µmol/L)/(TAS, mmol Trolox eq/L) × 100] [11].

### Genetic analysis

Reverse transcription and quantitative polymerase chain reaction (RT-qPCR) experiments began with RNA extraction from homogenized tissues using the GeneAll RiboEx™ RNA Isolation Kit (GeneAll Biotechnology, Seoul, Korea) according to the manufacturer’s instructions. The concentration and purity of the extracted RNA samples were measured using the BioSpec-nano nanodrop spectrophotometer (Shimadzu Ltd., Kyoto, Japan). For cDNA synthesis, 1 µg of RNA was used per sample. Complementary DNA (cDNA) was synthesized using the A.B.T.™ cDNA Synthesis Kit (Atlas Biotechnology, Turkey) in a thermal cycler following the kit protocol.

Primer sequences were designed by identifying specific mRNA targets and evaluating potential primers through the NCBI database. Primer sequences utilized in this study are available upon request. Gene expression levels were determined with the Biorad CFX96 real-time PCR system (Bio-Rad Laboratories, California, USA) using a 2X SYBR Green master mix (Nepenthe, Turkey). The GAPDH gene was employed as the internal control. Reaction mixtures were prepared to a final volume of 20 µl according to the master mix protocol. Real-time PCR was performed with the following cycling conditions: initial denaturation at 94 °C for 10 min (1 cycle), followed by 40 cycles of denaturation at 95 °C for 15 s, and annealing/extension at 55 °C for 30 s. Each sample was run in triplicate. Relative mRNA expression levels were calculated using the 2^ΔΔ^Ct method after normalization.

### Statistical analysis

Statistical analysis was performed using the GraphPad Prism program. Initially, the Shapiro-Wilk method was employed to assess the normality of the data distribution. ANOVA was used to compare the groups since the data showed a normal distribution (*p* > 0.05). Pairwise differences between the groups were obtained using the post hoc Tukey test. The significance threshold was set at *p* < 0.05, and the findings are shown as means ± standard error.

## Results

### Histopathological examination

**Fig. 1:**
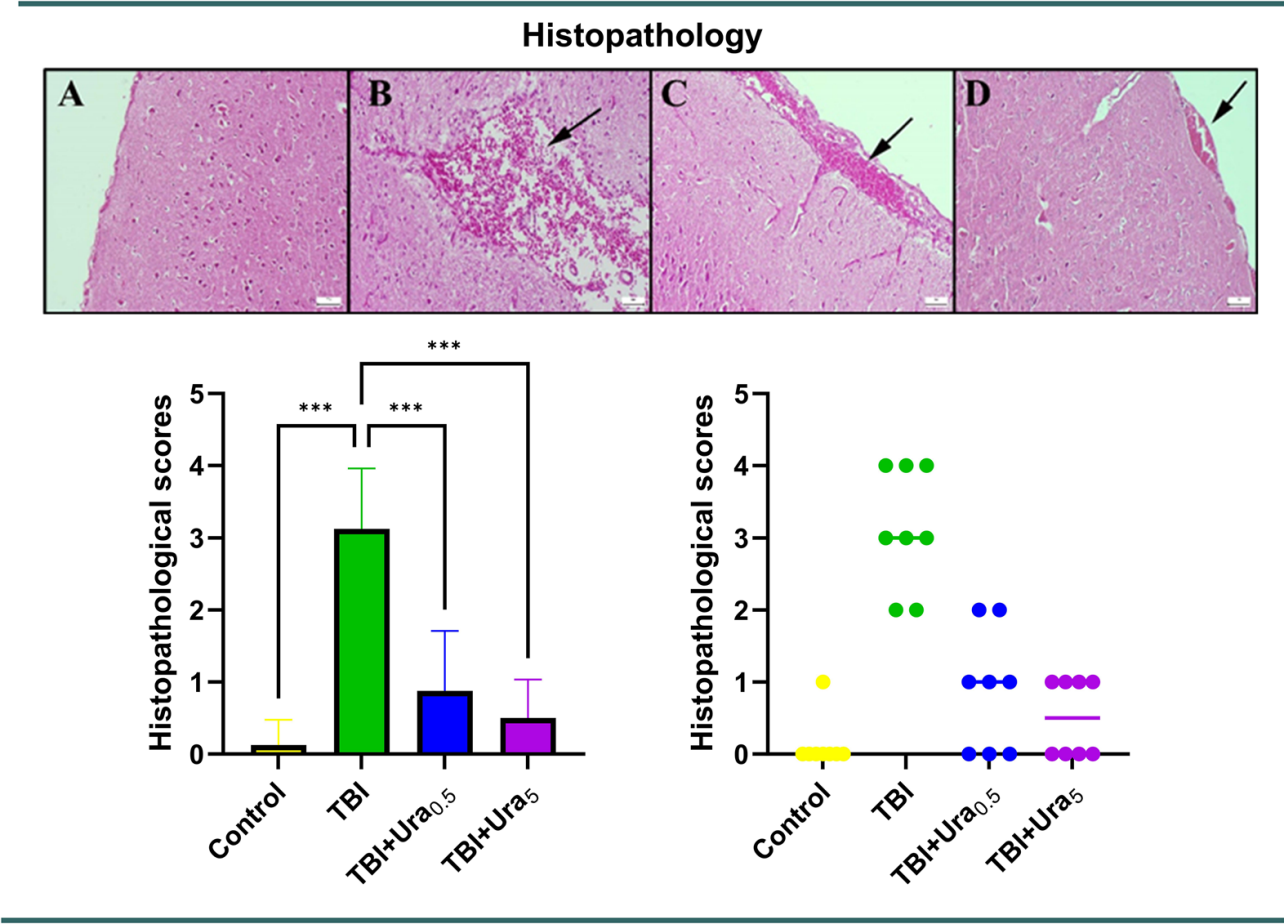
Histopathological appearance of brain tissues across the groups. (**A**) Control group, (**B**) TBI group, (**C**) TBI + Ura_0.5_ group, (**D**) TBI + Ura_5_ group. TBI: Traumatic brain injury, Ura_0.5_: Urapidil 0.5 mg/kg, Ura_5_: Urapidil 5 mg/kg. HE staining, scale bars = 50 μm

During the histological evaluation, normal brain histology was observed in the control group. The TBI group displayed extensive and pronounced hemorrhage (*p* < 0.001). Administration of Ura significantly reduced hemorrhagic regions in both the TBI + Ura_0.5_ and TBI + Ura_5_ mg groups (*p* < 0.001 for both). The treatment groups demonstrated a notable decrease and improvement in brain hemorrhage, with the TBI + Ura_5_ group being more effective than the TBI + Ura_0.5_ group (Fig. 1).

### Immunohistochemical findings

**Fig. 2:**
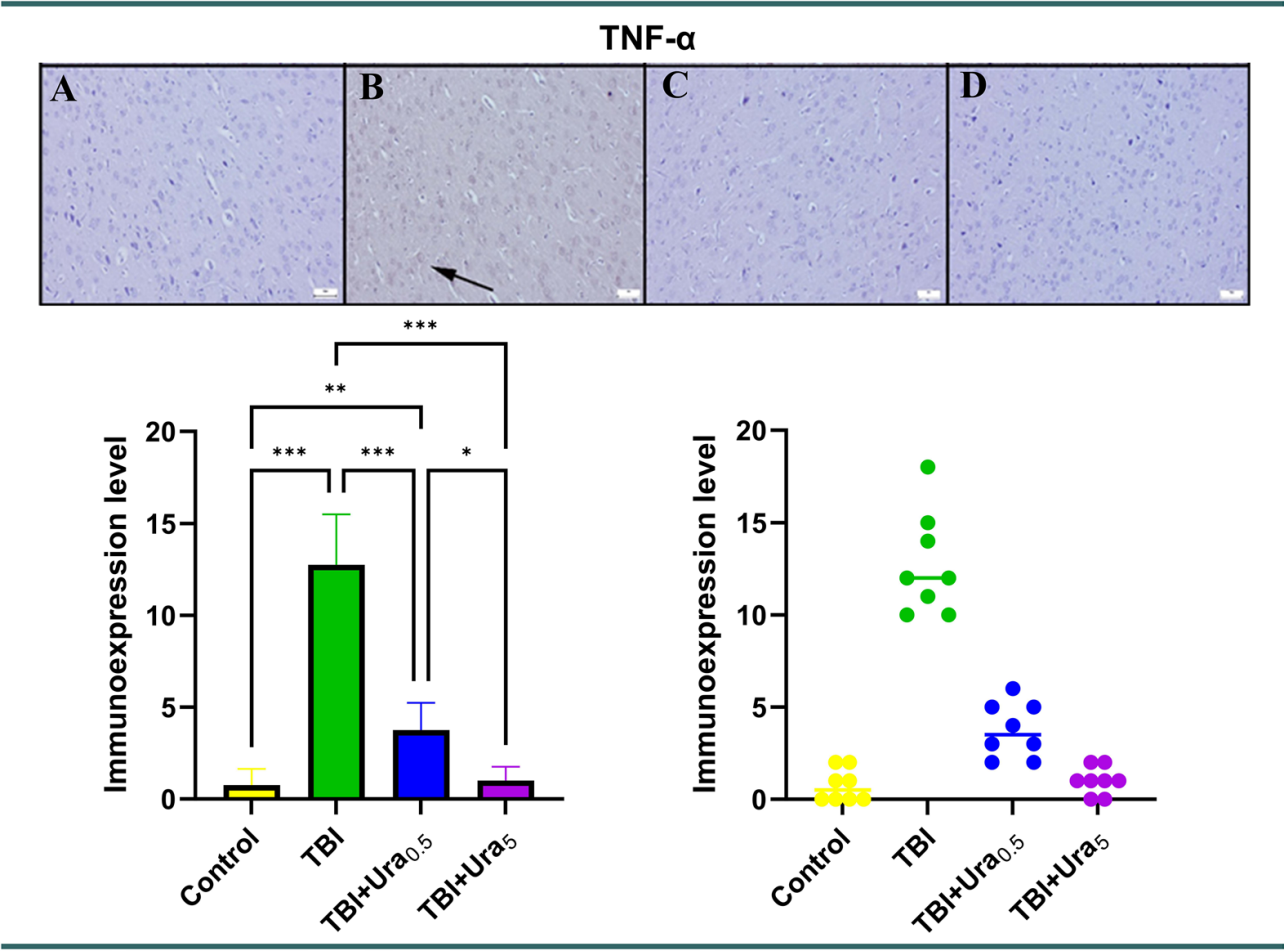
Expression of TNF-α in the brain. (**A**) Control group, (**B**) TBI group, (**C**) TBI + Ura_0.5_ group, (**D**) TBI + Ura_5_ group. TNF-α: Tumor necrosis factor-alpha, TBI: Traumatic brain injury, Ura_0.5_: Urapidil 0.5 mg/kg, Ura_5_: Urapidil 5 mg/kg. Streptavidin-biotin peroxidase method, scale bars = 50 μm

**Fig. 3:**
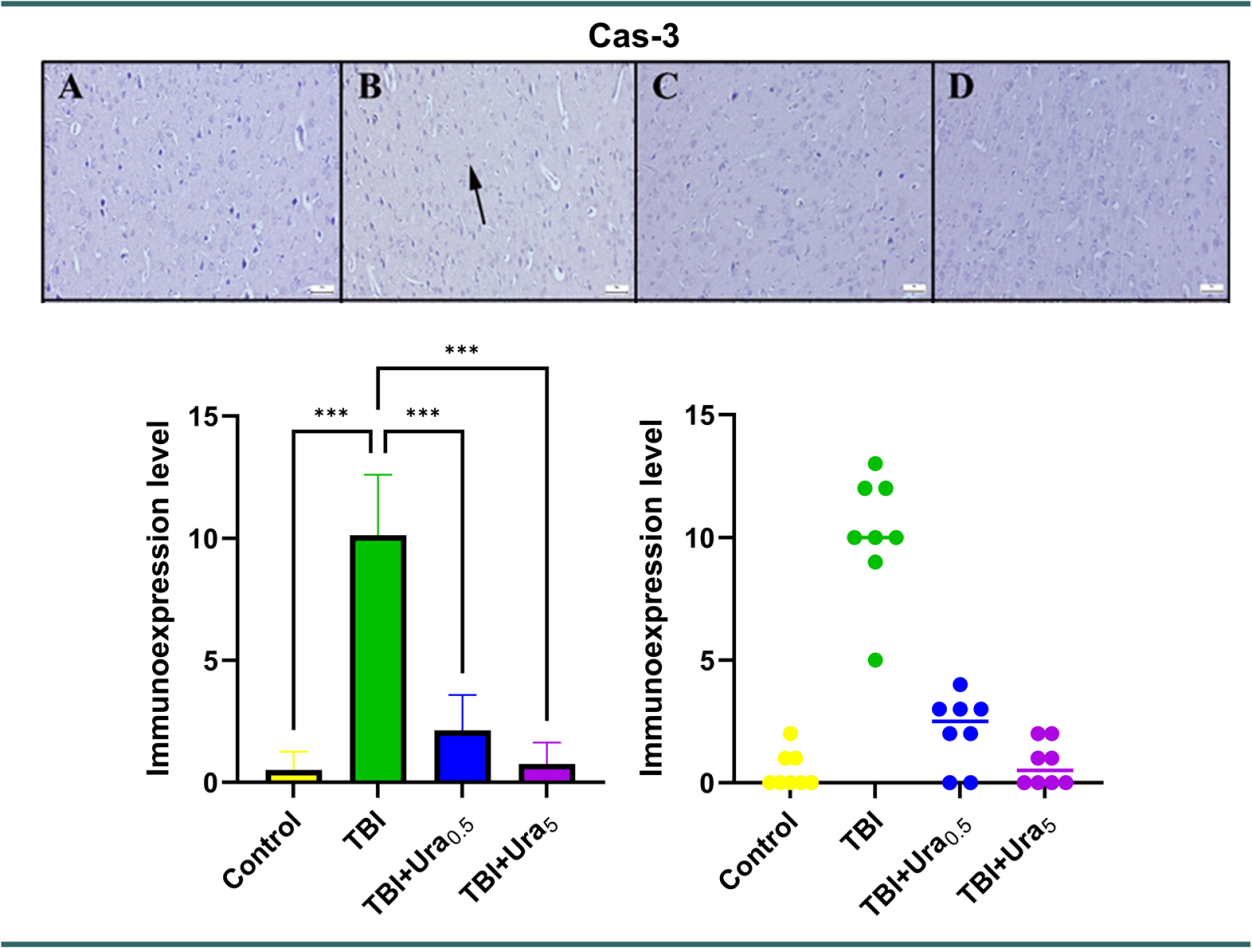
Expression of Cas-3 in the brain. (**A**) Control group, (**B**) TBI group, (**C**) TBI + Ura_0.5_ group, (**D**) TBI + Ura_5_ group. Cas-3: Caspase-3, TBI: Traumatic brain injury, Ura_0.5_: Urapidil 0.5 mg/kg, Ura_5_: Urapidil 5 mg/kg. Streptavidin-biotin peroxidase method, scale bars = 50 μm

**Fig. 4:**
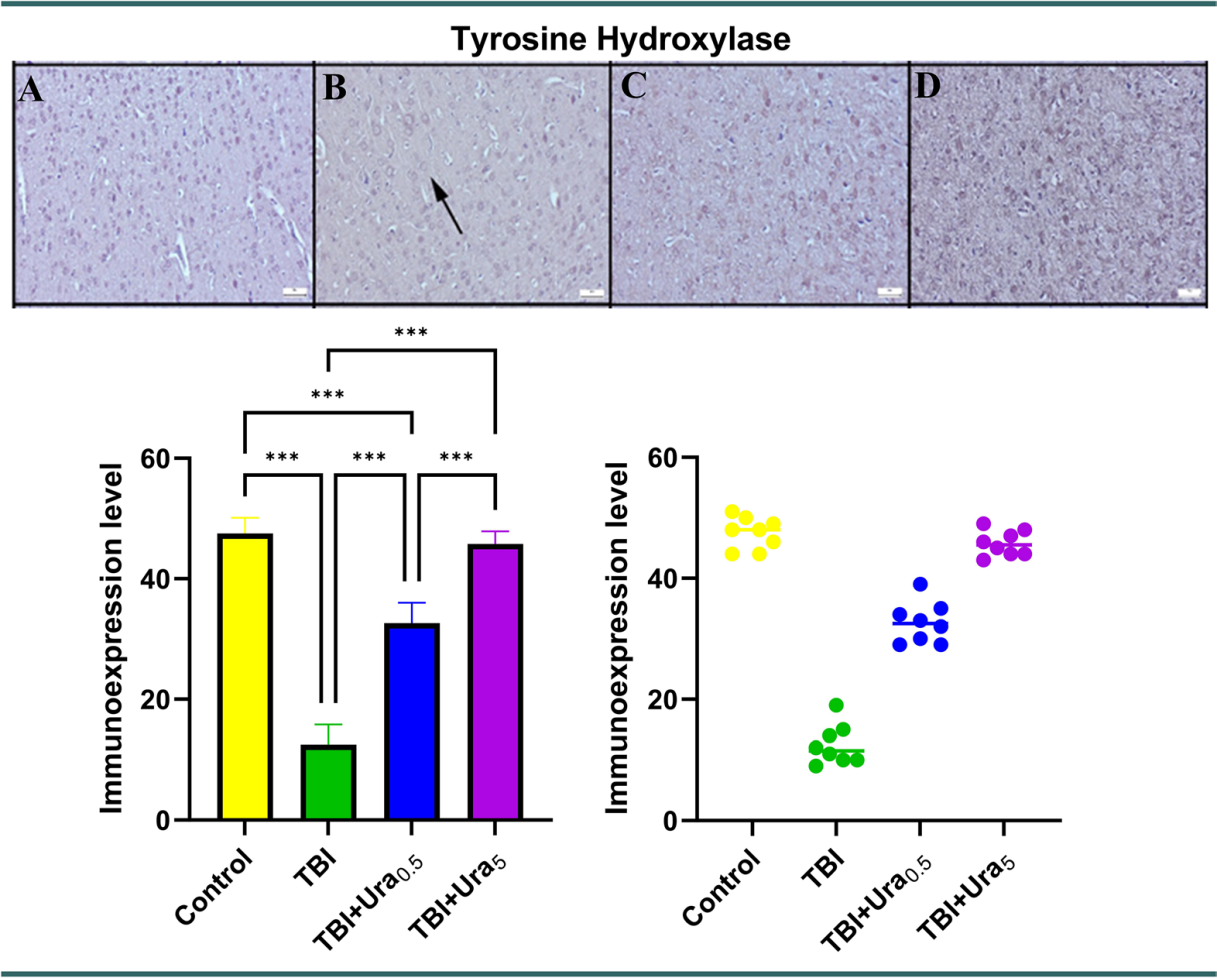
Expression of tyrosine hydroxylase in the brain. (**A**) Control group, (**B**) TBI group, (**C**) TBI + Ura_0.5_ group, (**D**) TBI + Ura_5_ group. TBI: Traumatic brain injury, Ura_0.5_: Urapidil 0.5 mg/kg, Ura_5_: Urapidil 5 mg/kg. Streptavidin-biotin peroxidase method, scale bars = 50 μm

Upon immunohistochemical examination, negative or slight Cas-3 and TNF-α expressions were observed, while distinct TH expressions were noted in the control group, The Tra group displayed mild to marked expression of Cas-3 and TNF-α, along with decreased expression of TH in the brains (*p* < 0.001 for all). However, Ura therapy reversed the expression of all markers in both the 0.5 mg and 5 mg groups, with the 5 mg group showing greater effectiveness than the 0.5 mg group (*p* < 0.001 for all). TH levels in TBI + Ura_0.5_ group were significantly lower compared to the control and TBI + Ura_5_ groups (*p* < 0.001 for both). TNF-α levels in TBI + Ura_0.5_ group were higher than the control and TBI + Ura_5_ group (*p* < 0.01 and *p* < 0.05, respectively) (Figs. 2, 3 and 4).

### Biochemical results

**Fig. 5:**
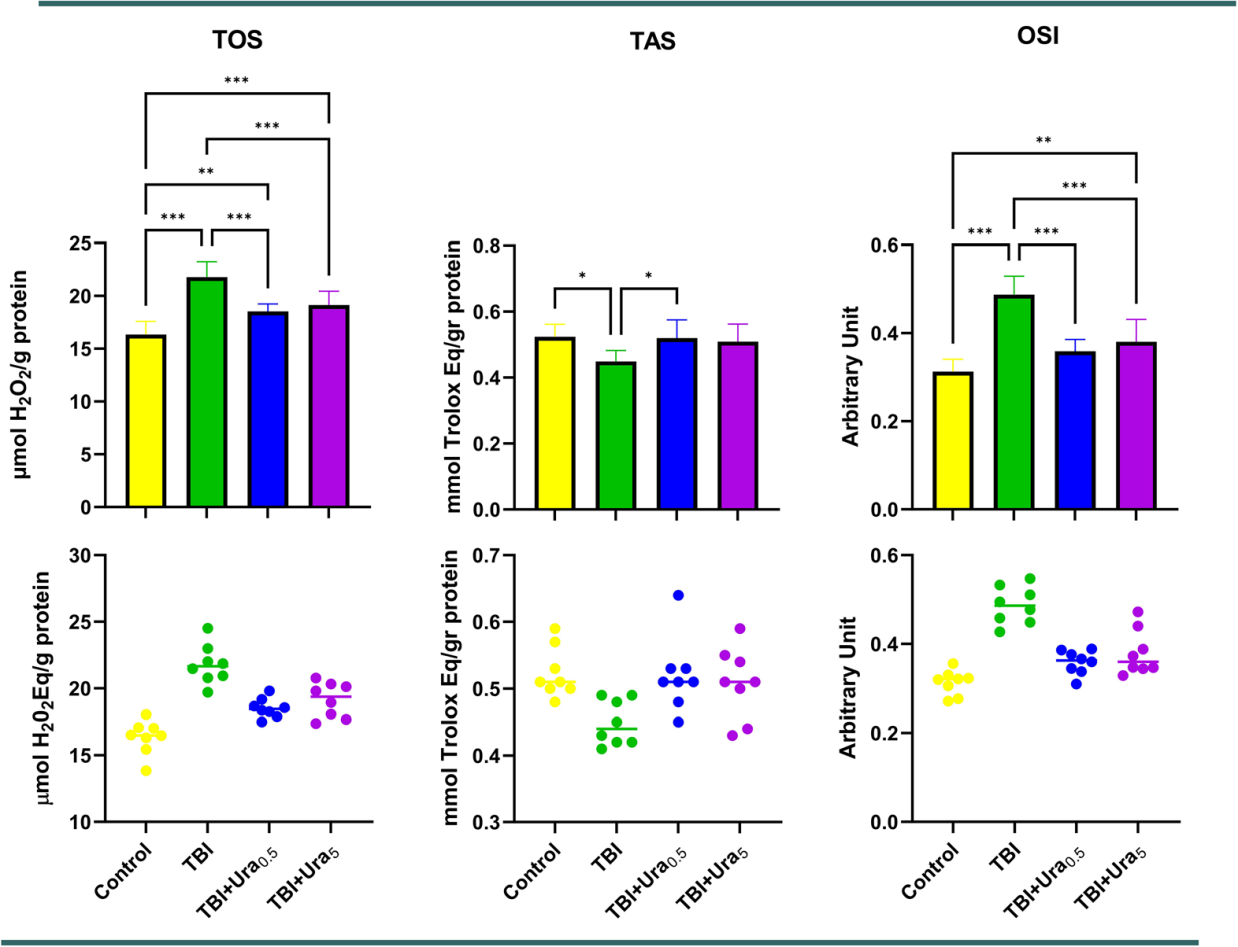
Oxidative stress parameters of brain tissues. Values are presented as means ± SD. The relationships between groups and results of biochemical markers are assessed by one-way ANOVA, post hoc Tukey’s test. TBI: Traumatic brain injury, Ura_0.5_: Urapidil 0.5 mg/kg, Ura_5_: Urapidil 5 mg/kg, TOS: Total oxidant status, TAS: Total antioxidant status, OSI: Oxidative stress index

To assess oxidative stress, TOS and TAS levels were measured, and OSI values were calculated. When comparing the control and TBI groups, the Tra group demonstrated significantly higher values for both TOS and OSI (*p* < 0.001 for both). Significant improvements in these two parameters were observed with both Ura treatments (*p* < 0.001 for all). Additionally, when the control group was compared to the TBI + Ura_5_ group, TOS levels remained significantly higher in the TBI + Ura_5_ group. No significant differences were observed in TAS results (Fig. 5).

### Genetic results

**Fig. 6:**
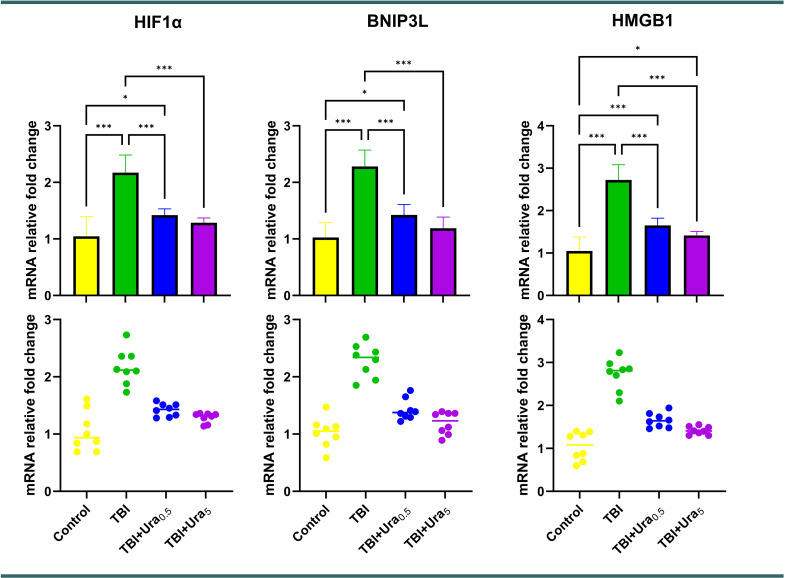
mRNA relative fold change of genes. Values are presented as means ± SD. The relationships between groups are assessed by one-way ANOVA, post hoc Tukey’s test. TBI: Traumatic brain injury, Ura_0.5_: Urapidil 0.5 mg/kg, Ura_5_: Urapidil 5 mg/kg, HIF1α: Hypoxia-Inducible Factor 1alpha, BNIP3L: BCL2 Interacting Protein 3-Like, HMGB1: High Mobility Group Box 1

Relative fold changes in mRNA expression levels of HIF1α, BNIP3L, and HMGB1 were evaluated in genetic analyses. The TBI group exhibited significantly higher expression levels for all three markers compared to the control group, whereas both treatment groups showed a significant reduction in these values (*p* < 0.001 for all) (Fig. 6).

## Discussion

TBI initiates a cascade of molecular and cellular responses, leading to progressive damage mediated by oxidative stress, inflammation, and apoptotic signaling pathways [12]. Our study evaluated the therapeutic potential of Ura in TBI, focusing on its effects on genetic, histopathological, and immunohistochemical markers. The results highlighted Ura’s capacity to mitigate secondary injury mechanisms, with particularly notable impacts on hypoxia, mitochondrial dysfunction, and inflammatory pathways as reflected in the genetic markers HIF1α, BNIP3L, and HMGB1.

HIF1α, a transcription factor activated by hypoxia, plays a dual role in TBI. While it promotes angiogenesis and cellular survival in low-oxygen conditions, its prolonged activation exacerbates neuroinflammation and apoptosis through the upregulation of inflammatory cytokines and pro-apoptotic genes [13, 14]. In our study, TBI-induced HIF1α upregulation was significantly reduced by Ura treatment, suggesting its ability to modulate the hypoxic response. This effect may contribute to the preservation of neuronal integrity by limiting hypoxia-driven secondary injury.

While the reduction in hemorrhagic burden observed in the Ura-treated groups may partially stem from its systemic hypotensive effect, this raises the theoretical concern of secondary hypoxia. However, both HIF-1α expression and histopathological evidence of hypoxia were reduced, suggesting that urapidil may improve oxygenation or reduce hypoxic stress through mechanisms beyond blood pressure lowering. Future studies incorporating hemodynamic monitoring will be necessary to clarify this complex interplay.

BNIP3L, a key regulator of mitophagy and apoptosis, is closely associated with mitochondrial health in neurons [15]. In TBI, BNIP3L overexpression disrupts mitochondrial membrane potential, triggering neuronal death and amplifying oxidative stress [16]. Ura significantly reduced BNIP3L expression in both treatment groups, indicating its potential to stabilize mitochondrial function. This finding aligns with the observed reductions in oxidative stress markers, such as TOS and OSI, further supporting the hypothesis that Ura mitigates mitochondrial dysfunction in TBI.

HMGB1, a nuclear protein released during cellular damage, acts as a pro-inflammatory mediator by engaging toll-like receptors (TLRs) and receptors for advanced glycation end-products (RAGE), amplifying inflammation and apoptosis [17, 18]. Elevated HMGB1 levels observed in the TBI group were substantially reversed by Ura, particularly at the higher dose. This reduction likely reflects Ura’s anti-inflammatory effects, potentially mediated through its modulation of systemic sympathetic responses and central serotonin receptor activity [7]. By decreasing HMGB1 levels, Ura may disrupt the inflammatory feedback loop, preventing further neuronal injury.

The genetic findings were corroborated by histopathological and immunohistochemical analyses. Extensive hemorrhage in the TBI group was significantly reduced in Ura-treated groups, particularly at the 5 mg dose. The reduction in hemorrhage likely reflects Ura’s vasodilatory effects, which improve microvascular perfusion and reduce vascular shear stress. These histological improvements align with reduced HIF1α expression, indicating alleviated hypoxia and improved vascular stability.

Immunohistochemical analyses further demonstrated Ura’s efficacy in modulating apoptosis and inflammation. Cas-3, a key executor of apoptosis, and TNF-α, a central pro-inflammatory cytokine, were significantly elevated in the TBI group. These markers were notably reduced in the treatment groups, reflecting suppressed apoptotic and inflammatory responses [19]. Meanwhile, TH, a marker of dopaminergic neuronal integrity, was restored in the Ura-treated groups, particularly in the 5 mg dose group. The correlation between restored TH levels and reduced BNIP3L expression underscores Ura’s role in preserving mitochondrial and neuronal function [20].

Biochemical assessments revealed that oxidative stress plays a pivotal role in TBI pathophysiology, as evidenced by elevated TOS and OSI levels in the TBI group. While TAS levels remained unchanged, Ura significantly reduced TOS and OSI, indicating its antioxidative properties. The reductions in oxidative stress markers align with suppressed BNIP3L and HMGB1 expression, suggesting a mechanistic link between mitochondrial stabilization, reduced inflammation, and improved oxidative balance.

The interplay between HIF1α, BNIP3L, and HMGB1 creates a self-reinforcing cycle of hypoxia, mitochondrial dysfunction, and inflammation in TBI [21]. Ura’s ability to downregulate these markers suggests a multifaceted mechanism of action, targeting multiple pathways simultaneously. By reducing hypoxia-driven inflammation, stabilizing mitochondrial function, and suppressing damage-associated molecular pattern molecules-mediated inflammatory responses, Ura interrupts the secondary injury cascade, ultimately preserving neuronal structure and function [22].

These findings observed in previous studies demonstrating Ura’s neuroprotective effects in other models of oxidative stress and inflammation. The integration of genetic, histopathological, and biochemical analyses in this study provides a comprehensive evaluation of Ura’s therapeutic potential. While our findings highlight urapidil’s neuroprotective effects in a rat model of TBI, further preclinical and clinical studies are warranted to explore its long-term efficacy and translational potential.

## Data Availability

The data that support the findings of this study are available from the corresponding author upon reasonable request.
